# Suppression of mRNAs Encoding Tegument Tetraspanins from *Schistosoma mansoni* Results in Impaired Tegument Turnover

**DOI:** 10.1371/journal.ppat.1000840

**Published:** 2010-04-15

**Authors:** Mai H. Tran, Tori C. Freitas, Leanne Cooper, Soraya Gaze, Michelle L. Gatton, Malcolm K. Jones, Erica Lovas, Edward J. Pearce, Alex Loukas

**Affiliations:** 1 Division of Infectious Diseases, Queensland Institute of Medical Research, Brisbane, Queensland, Australia; 2 Department of Pathobiology, School of Veterinary Medicine, University of Pennsylvania, Philadelphia, Pennsylvania, United States of America; 3 School of Veterinary Sciences, The University of Queensland, Brisbane, Queensland, Australia; Yale University, United States of America

## Abstract

Schistosomes express a family of integral membrane proteins, called tetraspanins (TSPs), in the outer surface membranes of the tegument. Two of these tetraspanins, *Sm*-TSP-1 and *Sm*-TSP-2, confer protection as vaccines in mice, and individuals who are naturally resistant to *S. mansoni* infection mount a strong IgG response to *Sm*-TSP-2. To determine their functions in the tegument of *S. mansoni* we used RNA interference to silence expression of *Sm*-*tsp-1* and *Sm*-*tsp-2* mRNAs. Soaking of parasites in *Sm-tsp* dsRNAs resulted in 61% (p = 0.009) and 74% (p = 0.009) reductions in *Sm*-*tsp-1* and *Sm*-*tsp-2* transcription levels, respectively, in adult worms, and 67%–75% (p = 0.011) and 69%–89% (p = 0.004) reductions in *Sm*-*tsp-1* and *Sm*-*tsp-2* transcription levels, respectively, in schistosomula compared to worms treated with irrelevant control (*luciferase*) dsRNA. Ultrastructural morphology of adult worms treated *in vitro* with *Sm*-*tsp-2* dsRNA displayed a distinctly vacuolated and thinner tegument compared with controls. Schistosomula exposed *in vitro* to *Sm*-*tsp-2* dsRNA had a significantly thinner and more vacuolated tegument, and morphology consistent with a failure of tegumentary invaginations to close. Injection of mice with schistosomula that had been electroporated with *Sm*-*tsp-1* and *Sm*-*tsp-2* dsRNAs resulted in 61% (p = 0.005) and 83% (p = 0.002) reductions in the numbers of parasites recovered from the mesenteries four weeks later when compared to dsRNA-treated controls. These results imply that tetraspanins play important structural roles impacting tegument development, maturation or stability.

## Author Summary

Schistosomes, or blood flukes, reside in the blood vessels surrounding the liver and bowel of their human hosts. They infect 200 million people and kill many thousands each year in developing countries. The parasites cover themselves in a unique series of cell membranes called the tegument. Molecules in the tegument membranes are a major target for the development of new drugs and vaccines against the parasite. Here we show that at least one member of a family of tegument membrane proteins called tetraspanins, *Sm*-TSP-2, is integral to the proper formation of the tegument and subsequent survival of the parasite in its human host, providing a potential mechanism by which a vaccine based on *Sm*-TSP-2 protects immunized hosts.

## Introduction

Schistosomes are parasitic trematodes that cause chronic infection in over 207 million people in 76 developing tropical countries. Schistosomiasis is generally associated with poverty, poor water supply and inadequate sanitation [1]. Infection rates and intensities are high in early childhood, peak around 8 to 15 years and decrease in adulthood [2]. Despite effective and inexpensive widespread treatment with the anthelmintic drug praziquantel for over 20 years, this parasitic disease still causes more than 250,000 deaths per year and accounts for 1.7 to 4.5 million disability-adjusted life years (DALYs) lost annually [3].

Humans become infected with schistosomes when they are exposed to free-living cercariae in fresh water. Cercariae penetrate the skin, shed their tails and transform into schistosomula, which reside in the dermis of the skin before entering the blood capillaries to migrate through the vasculature to the portal venous system where they mature into adult worms [4]. The outer surface of schistosomula and adult worms, the tegument, is a multinucleated syncitium that contains tegumental cell bodies situated below the muscular layers. During transformation from cercaria to schistosomula, the outer surface of the tegument (the interface with the host) is remodeled from a single membrane with a prominent glycocalyx into an unusual double membrane (or “heptalaminate”) structure [5]. This double membrane is widely believed to play an essential role in the ability of schistosomes to evade the host immune system, a characteristic that allows them to live for years within their hosts [6]. The outer of the two surface membranes also has the ability to adsorb host blood molecules, masking its non-self status thereby contributing to immune evasion and prolonged survival [7]. We believe that tegumental proteins are ideal targets for immunological and pharmacological intervention [8]. The generation of a large number of *S. mansoni* expressed sequence tags [9] and the recently completed genome sequence [10], in combination with advances in characterizing the tegument proteome has led to the discovery of many tegument specific proteins [11]. Among them are a group of membrane proteins called tetraspanins, which are highly expressed in the outer tegument membrane of adult schistosomes [12], [13]. To date, five tetraspanin cDNAs have been described from *S. mansoni*, namely *Sm-23* [12], *Sm-tsp-1* and *Sm-tsp-2* [13], *Sm-tetraspanin-B* and *Sm-tetraspanin-C* [14].

Tetraspanins are a large superfamily of surface-associated membrane proteins characterized by the conserved structure of four hydrophobic transmembrane domains, a small and large extracellular loop, an interconnecting intracellular loop, and cytoplasmic amino- and carboxyl- termini [15]. Tetraspanins undergo post-translational modification in which palmitate is bound to the membrane proximal cysteine residues and associates with cholesterol-rich domains [16]. This process enables tetraspanins to play key roles in molecular organization of cell membranes, interacting with one another and also specific partner proteins such as integrins, MHC and co-stimulatory molecules to form large signal transducing complexes termed tetraspanin-enriched microdomains (TEMs) [17]. Tetraspanins are widely distributed in many cell types but their physiological roles are mostly unknown. Several lines of evidence have implicated tetraspanins in the regulation of cell adhesion, differentiation, motility, aggregation, cell signaling and sperm-egg fusion [18], [19], [20], [21]. They have been linked to various pathological processes including lymphocyte activation [19], cancer [22], fertilization [23], [24], and interactions between pathogens and host cells such as HIV [25], HCV [26] and *Plasmodium* [27].

We previously identified two cDNAs, *Sm-tsp-1* (Sm01494) and *Sm-tsp-2* (Sm12366), in adult *S. mansoni* using signal sequence trapping [13], and showed that both of these tetraspanins were expressed in the tegument of the adult parasite [28]. Other authors confirmed the surface expression of these tetraspanins using various mass spectrometric approaches to characterize the schistosome surface [11], [29], [30]. We expressed the large extracellular loop of *Sm*-TSP-1 and *Sm*-TSP-2 in *E. coli* and used the soluble recombinant proteins to immunize mice and then challenged them with cercariae. Mice vaccinated with recombinant *Sm*-TSP-1 and *Sm*-TSP-2 had significantly reduced adult worm, liver egg and fecal egg burdens [28]. Moreover, strong IgG1 and IgG3 antibody responses against *Sm*-TSP-2 were detected in sera of individuals deemed putatively resistant (PR) to *S. mansoni* in comparison to sera from chronically infected individuals [28].

Despite their promise as vaccines against schistosomiasis, the functions of *Sm*-TSP-1 and *Sm*-TSP-2 have not yet been elucidated. We therefore employed RNA interference (RNAi) to explore the roles of *Sm-tsp-1* and *tsp-2* in larval and adult *S. mansoni*. RNAi has been utilized with *S. mansoni* to suppress endogenous gene expression in schistosomula [31], adult worms [32], eggs [33] and sporocysts [34]. Here, we show that RNAi results in reductions in expression of *Sm-tsp-1* and *tsp-2* mRNAs in schistosomula and adult worms, and malformation of the tegument in worms cultured *in vitro*. Moreover, silencing of *tsp-1* and *tsp-2* expression in schistosomula results in up to 90% fewer worms maturing to adulthood when introduced into mice compared with parasites exposed to control dsRNAs, highlighting their essential roles in tegument biogenesis and maintenance and further supporting the development of novel therapies targeting these genes and their protein products.

## Results

### Developmental expression of *Sm-tsp-1* and *Sm-tsp-2* in *S. mansoni*

**Figure 1:**
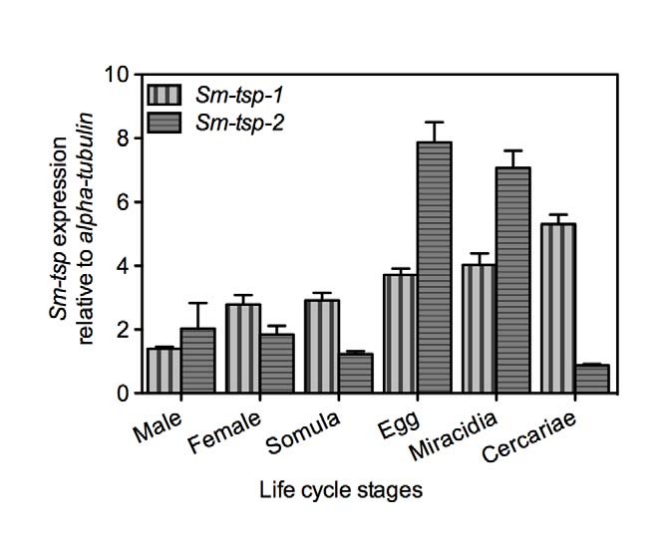
Expression of *Sm*-*tsp-1* and *Sm-tsp-2* at different stages of the *S. mansoni* life cycle. RNA levels of *Sm-tsp-1* (vertical bars) and *Sm-tsp-2* (horizontal bars) relative to *Sm*-α-*tubulin* were analyzed by qRT-PCR. Data are representative of mean±S.E. from three separate experiments.

Expression of *Sm-tsp-1* and *Sm-tsp-2* mRNAs in different stages of the *S. mansoni* life cycle was determined relative to control *Sm*-α-*tubilin* mRNA using qRT-PCR. *Sm-tsp-1* and *Sm-tsp-2* mRNAs were detected in all stages of the schistosome life cycle with higher levels identified in eggs, miracidia and cercariae than in 5-day old schistosomula, males and female worms for *tsp-1*; a similar expression profile was observed for *tsp-2* but gene expression was notably reduced in cercariae (Figure 1). Interestingly, the highest level of *Sm-tsp-1* expression was detected in cercariae whereas *Sm-tsp-2* expression was lowest in cercariae.

### Expression of *Sm*-TSP-1 and *Sm*-TSP-2 in the tegument of schistosomula

**Figure 2:**
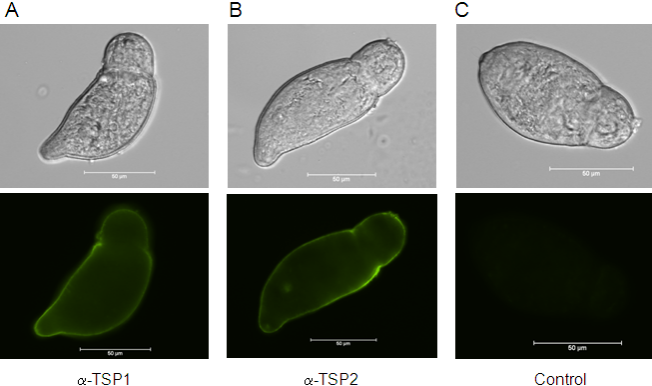
Expression of *Sm*-TSP-1 or *Sm*-TSP-2 on the surface of live schistosomula. Immunofluorescent labelling of live 3 h schistosomula with antisera raised against *Sm*-TSP-1 (A) and *Sm*-TSP-2 (B), and control pre-immune serum (C) followed by anti-mouse Ig-FITC. Schistosomula are shown in bright-field and FITC stained. Scale = 50 µm.

We previously demonstrated that *Sm*-TSP-1 and *Sm*-TSP-2 are expressed on the tegument surface membrane of adult worms [28]. The tegument is fully formed by 3h after cercarial transformation [35], so to determine whether these TSPs are expressed in the tegument at this early stage after host entry and whether they are accessible to antibodies on live parasites, we probed live newly transformed schistosomula with antibodies against both proteins. Both *Sm*-TSP-1 and *Sm*-TSP-2 were detected over the entire surface tegument of live schistosomula when probed with mouse anti-TSP-1 or -TSP-2 sera followed by FITC-labelled anti-mouse IgG (Figure 2).

### dsRNA-mediated knockdown of *Sm-tsp* expression in adult worms

**Figure 3:**
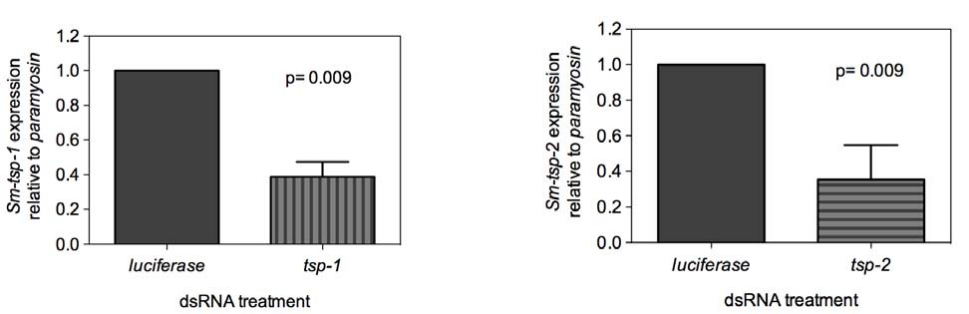
Suppression of *Sm-tsp* mRNAs in adult parasites by RNAi. *Sm-tsp-1* (A) and *Sm-tsp-2* (B) transcript levels relative to *Sm*-*paramyosin* (mean±S.E.) in adult parasites soaked for 7 days with 1 µg/ml of *Sm-tsp* or *luciferase* control dsRNAs.

Adult worms soaked for 7 days in *Sm*-*tsp-1* dsRNA had a 61% (p = 0.009) reduction in *Sm-tsp-1* mRNA expression compared to parasites soaked in control dsRNA (Figure 3A). A 74% (p = 0.009) reduction in *Sm-tsp-2* mRNA levels was detected in worms that were cultured in media containing *Sm*-*tsp-2* dsRNA compared to parasites soaked in *luciferase* dsRNA (Figure 3B). Parasites were visually monitored for motility on a daily basis but no differences were detected between groups (not shown).

### dsRNA-mediated knockdown of *Sm-tsp* expression in schistosomula

**Figure 4:**
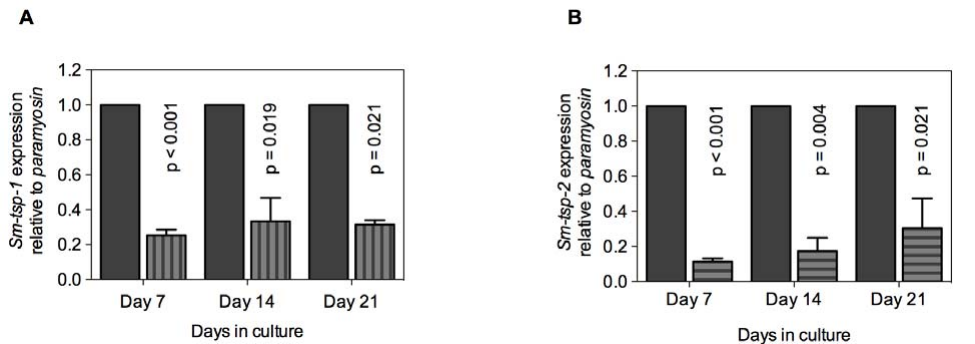
Suppression of *Sm-tsp* mRNAs in schistosomula by RNAi. *Sm-tsp-1* (A) and *Sm-tsp-2* (B) transcript levels relative to *Sm-paramyosin* (mean±S.E.) in schistosomula soaked for 7, 14 and 21 days with 1 µg/ml of *Sm-tsp* or *luciferase* control dsRNAs.

Soaking of 3 h old schistosomula in *Sm-tsp-1* dsRNA for 7, 14 and 21 days caused 75% (p<0.001), 67% (p = 0.019) and 69% (p = 0.021) decreases in *Sm*-*tsp-1* mRNA expression in comparison to the control group (Figure 4A). Larval parasites incubated with *Sm*-*tsp-2* dsRNA for 7 days exhibited an 88% (p<0.001) decrease in *Sm-tsp-2* transcript levels compared to *luciferase* dsRNA treated schistosomula (Figure 4B). RNAi knockdown was maintained with reductions of 82% (p = 0.004) and 69% (p = 0.021) at days 14 and 21, respectively, compared to the control group. As observed in adult worms, suppression of *Sm-tsp* RNAs resulted in no obvious phenotypic differences compared to the luciferase dsRNA-treated control group when examined by light microscopy. Cultures were visually inspected using a light microscope on a daily basis and no differences in early growth and development of schistosomula (development of intestinal ceca or size of schistosomula) [36] were apparent between test and control dsRNA treated groups.

### Reduction in *Sm*-TSP2 protein expression in parasites treated with *Sm-tsp-2* dsRNA

**Figure 5:**
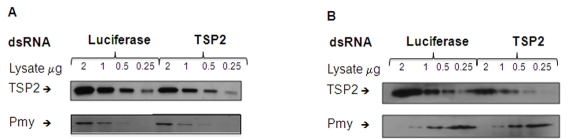
Protein expression levels of parasites treated with *Sm-tsp-2* dsRNA. Protein extracts from adult parasites (A) and schistosomula (B) treated with *Sm-tsp-2* or *luciferase* dsRNAs for 7 days were loaded onto a 12% SDS-PAGE gel at 2, 1, 0.5 and 0.25 µg. Proteins were transferred onto nitrocellulose and immunoblotted with anti-*Sm*-TSP2 or anti-*Sm*-Pmy monoclonal antibodies. The intensity of paramyosin expression was evaluated to determine equal protein loading.

To determine whether knockdown of *Sm*-*tsp-2* RNA was evident at the protein level, we performed Western blot analysis on dsRNA treated adult (Figure 5A) and larval (Figure 5B) parasites. Parasites were treated with *Sm*-*tsp-2* or *luciferase* dsRNAs, lysed in 1% Triton X-100 and immunoblotted with anti-*Sm*-TSP-2 or anti-*Sm*-Pmy antibodies which target a sub-tegumental muscle protein, paramyosin [37]. *Sm*-TSP-2 protein expression was decreased in adult worms treated with *Sm-tsp-2* dsRNA compared to worms treated with *luciferase* dsRNA for the four concentrations (2.0, 1.0, 0.5 and 0.25 µg) tested. In contrast, the *Sm*-Pmy protein expression levels did not change in both test and control groups. The experiment was repeated three times with similar results and a representative image is shown (Figure 5A). Densitometry analysis was performed on each band and the ratio of *Sm*-TSP-2 to *Sm*-Pmy at each concentration was calculated. Analysis of whole worm lysates (0.25 µg) by densitometry (not shown) revealed an average of 61% (p = 0.027) reduction in *Sm*-TSP-2 expression in adult worms treated with *Sm-tsp-2* dsRNA compared to the control *luciferase* group. For RNAi treated schistosomula, the amount of *Sm*-TSP-2 protein expressed by schistosomula after 7 days in culture with *Sm-tsp-2* dsRNA was reduced compared to parasites soaked in *luciferase* dsRNA (Figure 5B). Densitometry analysis of lysates (2 µg, 1 µg and 0.5 µg) showed an average decline of 36% (data not shown). This decrease was lower than expected since suppression of *Sm-tsp-2* mRNA was more pronounced in schistosomula than in adult parasites. Adult and larval parasites soaked in *Sm-tsp-1* dsRNA demonstrated no obvious differences in protein expression to *luciferase* dsRNA control worms by Western blotting analysis (data not shown).

### Suppression of *Sm-tsp-2* mRNA results in malformation of the tegument when observed using transmission electron microscopy

**Figure 6:**
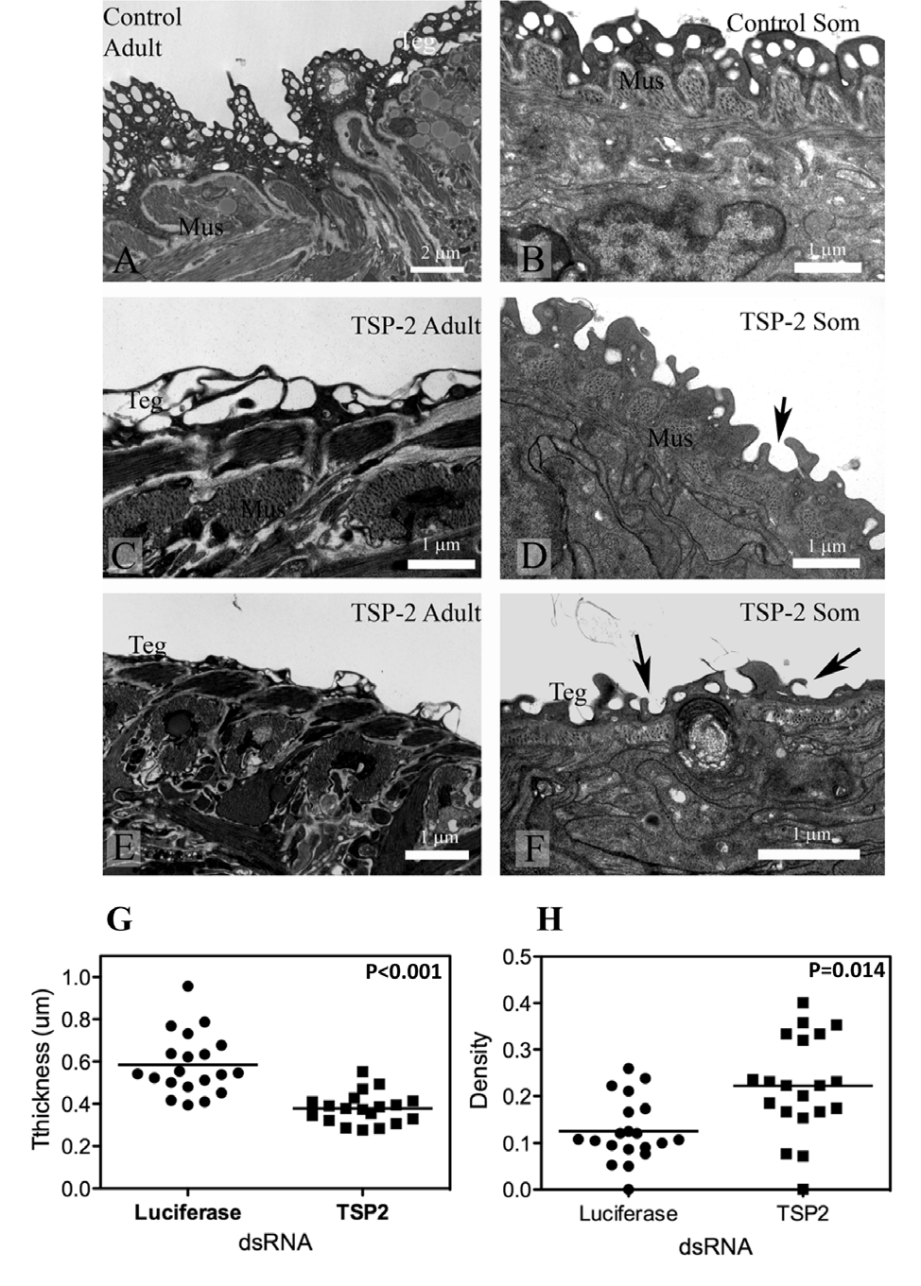
Ultrastructure of the tegument of parasites treated with *Sm-tsp-2* dsRNA RNA observed using transmission electron microscopy. A. Tegument of adult female treated with *luciferase* dsRNA. B. Tegument of schistosomulum incubated for 7 days with *luciferase* dsRNA. C and E. Tegument of adult female incubated with *Sm-tsp-2* dsRNA. The tegument is more highly vacuolated (C) and thinner (E) compared with controls. D and F. Tegument of schistosomula incubated for 7 days with *Sm-tsp-2* dsRNA. Digitate extensions (arrows) are more abundant on the surface of the tegument. Abbreviations: Mus-muscles; teg-surface layer of tegument. The tegument of schistosomula were thinner, p<0.001 (G) and more dense, p = 0.014 (H) in *Sm-tsp-2* dsRNA treated schistosomula.

Adult parasites and schistosomula treated with *Sm-tsp-2* dsRNA *in vitro* displayed modified tegument structure when visualized with transmission electron microscopy (TEM) compared with *luciferase* dsRNA treated controls (Figure 6). The tegument of adult worms incubated *in vitro* in *Sm-tsp-2* dsRNA (Figure 6C,E) was more highly vacuolated than *luciferase* dsRNA controls (Figure 6A), with extensive and enlarged vacuoles throughout the surface layer. The tegument of these parasites had less apparent cytoplasm and hence fewer cytoplasmic inclusions and was frequently much thinner than that of controls (Figure 6C,E). Schistosomula transformed and cultured *in vitro* presented a tegument that resembled that of larvae from natural or experimental infection (Figure 6B) [38]. The tegument in *Sm-tsp-2* dsRNA treated schistosomula (Figure 6D,F) was consistently thinner than those of luciferase controls (P<0.001), measuring on average 0.3784±0.016 µm compared with 0.5842±0.323 µm for luciferase controls (Figure 6G). Volume density measures for invaginations and clear vesicular compartments of the tegument showed higher volumes for these compartments in *Sm-tsp-2* treated schistosomula (p = 0.014; Figure 6F). The morphology of the schistosomula tegument was consistent with a failure to close invaginations of the surface (Figure 6D,F). Adult worms and schistosomula soaked in *Sm-tsp-1* dsRNA showed no obvious differences to *luciferase* dsRNA control worms when examined by transmission electron microscopy (data not shown).

### Suppression of *Sm-tsp* mRNAs in schistosomula affects parasite survival *in vivo*

**Figure 7:**
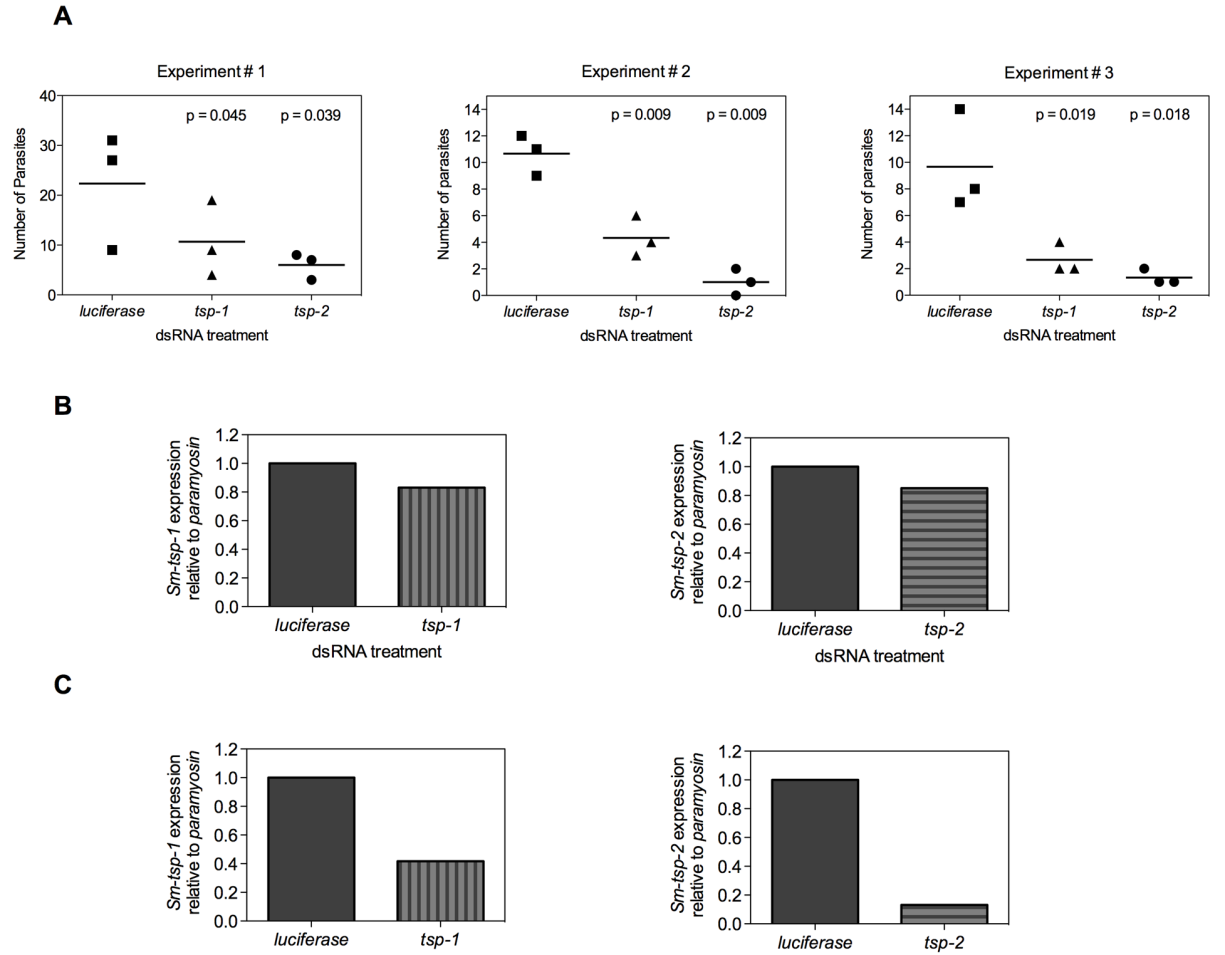
Infection of mice with *Sm-tsp* dsRNA treated schistosomula. Schistosomula were electroporated with 100 µg/ml of *Sm-tsp-1*, *Sm-tsp-2* or *luciferase* dsRNAs, washed and counted. C57BL/6 female mice were immunized intramuscularly with 2,000 dsRNA treated schistosomula and were perfused 4 weeks later to determine parasite numbers (A). Expression of *Sm-tsp-1 and Sm-tsp-2* mRNA transcript levels of parasites harvested from Experiment 1 (B). The *Sm-tsp-1* and *Sm-tsp-2* transcript levels of schistosomula that were electroporated and concurrently cultured *in vitro* for 4 weeks were also determined (C).

In the mammalian host, larval schistosomes migrate from the skin through the lungs to the liver and then mature in the mesenteric veins [4]. In an effort to mimic *in vivo* conditions, 3 h schistosomula were electroporated with 100 µg/ml of *Sm-tsp-1*, *Sm-tsp-2* or *luciferase* dsRNA and then injected intramuscularly into female C57BL/6 mice. Four weeks later mice were perfused to determine the number of parasites that reached maturity in the mesenteries. Significantly fewer parasites were recovered from the mesenteric veins compared to the *luciferase* control group (see Figure 7A for results of three experiments). Mice injected with schistosomula that were electroporated with *Sm-tsp-1* dsRNA yielded 48% (p = 0.045), 60% (p = 0.009) and 67% (p = 0.019) reduction in the number of parasites recovered for Experiments 1, 2 and 3, respectively in comparison to the *luciferase* control group. Schistosomula pretreated with *Sm-tsp-2* dsRNA and then injected into mice resulted in 70% (p = 0.039), 91% (p = 0.009) and 78% (p = 0.018) decreases in parasite survival for Experiments 1, 2 and 3, respectively when compared to the *luciferase* dsRNA group. The numbers of mature worms harvested from the *luciferase* control group were very low, with recovery ranging from 0.5-1.5%, however the data was consistent between three experiments, with a reproducible and significant reduction in worm recovery rates between *tsp* and *luciferase* dsRNA treated parasites.

RNA was extracted from surviving worms that were perfused from mice and transcript levels were analyzed by qRT-PCR. *Sm-tsp-1* expression was only slightly lower (17%) in worms recovered from mice that were infected with *Sm-tsp-1* dsRNA-treated schistosomula compared to the control group. Likewise, *Sm-tsp-2* expression was slightly reduced (15%) in worms recovered from mice that were infected with *Sm-tsp-2* dsRNA treated worms compared to the *luciferase* control group (Figure 7B). However, when the same batch of dsRNA electroporated schistosomula were cultured *in vitro* for the same period of time (4 weeks), as opposed to being injected into mice, significant knockdown of *Sm-tsp-1* and *Sm-tsp-2* transcripts by 58% and 87%, respectively (Figure 7C), was observed. These results illustrate that silencing of *Sm-tsp-1* and *Sm-tsp-2* by either soaking or electroporation leads to suppression of tetraspanin genes in schistosomes, and suppression is maintained for at least 4 weeks in culture. The data also implies one of three possible outcomes for *Sm-tsp* dsRNA treated schistosomula that survived to adulthood after being transferred into mice; (1) RNAi was not as effective in those individual schistosomula that survived in mice as opposed to those that perished; (2) some of the RNAi treated parasites received (or took up) less dsRNA, and therefore the efficacy of gene suppression was variable between individuals in a single electroporated batch; (3) it is also possible that host developmental cues stimulate transcription.

## Discussion

Schistosomes express a family of tetraspanins in their tegument. Sm23 was the first tetraspanin identified in *S. mansoni* [12], and is of interest as a DNA vaccine antigen against schistosomiasis [39]. Its orthologue from *S. japonicum*, Sj23, protects water buffaloes against challenge infection when administered as a DNA vaccine [39]. We identified two additional tetraspanins, *Sm-tsp-1* and *Sm-tsp-2*, which showed high levels of protection when administered to mice as recombinant protein vaccines against *S. mansoni* [13], [28]. However, despite the protective efficacy that these tetraspanins afford, their functions in the parasite are unknown. To understand the roles that these proteins play in the schistosome tegument, we herein explored the effects of silencing the expression of *Sm-tsp-1 and Sm-tsp-2* mRNAs in adult and larval *S. mansoni*.

RNAi has been used to suppress a number of schistosome genes in an effort to determine their functions [40], [41]. Soaking of *S. mansoni* with dsRNA encoding the intestinal protease cathepsin B (SmCB1), resulted in greater than 10-fold decrease in SmCB1 mRNA levels and significant growth inhibition compared to parasites treated with control dsRNA [42]. Suppression of the mRNA encoding another intestinal protease, *S. mansoni* cathepsin D (SmCD), in schistosomula by electroporation with dsRNA led to reduction in RNA transcript levels, growth retardation *in vitro* and *in vivo*, and decreased cathepsin D enzymatic activity [43]. Silencing of the SmAQP gene encoding a water channel protein by electroporating schistosomula with short interfering RNAs suppressed mRNA and protein expression in the tegument, and treated parasites cultured *in vitro* exhibited stunted growth and lower viability [44]. RNAi has been used to determine the functional importance of tetraspanins in other organisms [45]. Suppression of *tetraspanin-15* mRNA by feeding *C. elegans* with dsRNA resulted in dissociation of the cuticle and degeneration of the hypodermis, compromising epidermal integrity [46]. RNAi has also been used to determine the function of human tetraspanins in various cell types [45]. For example, the CD151 tetraspanin interacts with membrane proteins including the laminin-binding integrin α3β1; when lung adenocarcinoma cells were cultured on laminin-511 and then treated with CD151 siRNA, abnormal membrane protrusions on laminin-511 were apparent and tyrosine phosphorylation dependent signalling was reduced [47]. These findings indicate a role for tetraspanins in the maintenance of cell membrane biogenesis and structural integrity, and support our observations on the compromised tegument membrane formation in *S. mansoni* when *tsp* mRNA expression is suppressed.

Numerous reports have documented molecular interactions between tetraspanins and MHC, and involvement of human tetraspanins in regulating T cell co-stimulation and peptide/MHC presentation [48], [49], [50], indicating additional, non-structural roles. Schistosomes acquire host MHC onto their surfaces [51], presenting the intriguing possibility that they function as a receptor for host MHC. However, the majority of mammalian tetraspanin binding partners identified to date are membrane proteins rather than extracellular ligands [45]; moreover, our data presented here implies that schistosome tetraspanins are pivotal for proper tegument formation, even during *in vitro* culture in the absence of immune cells, supporting a structural role in the establishment and maintenance of the tegument. Indeed, the tetraspanin CD9 complexes with numerous proteins including Ig-containing proteins [52], a family of proteins which are also present in the *S. mansoni* tegument membrane [30]. Various authors have described the contribution of tetraspanins, such as CD9 and CD151, with members of the integrin family in promoting cell-cell interactions and migration [53], [54], [55]. Mass spectrometric analysis of the *S. mansoni* tegument revealed a β-integrin subunit in the sub-tegumental layer [29]. Suppression of tetraspanin mRNA expression in schistosomes may affect lateral interactions with integrins in the tegument, and the parasite's ability to migrate through the lungs to the liver and mesenteries where they would mature. The binding partner(s) associated with *Sm*-TSP-1 or *Sm*-TSP-2, or any of the other three *S. mansoni* tegument tetraspanins, have yet to be identified. We have produced monoclonal antibodies to *Sm*-TSP-2 and these antibodies are being used to immunoprecipitate *Sm*-TSP-2 and its binding partners in an effort to unravel the tegumental tetraspanin web.

To assess the viability of dsRNA treated parasites *in vivo*, we injected *tsp* or *luciferase* dsRNA treated parasites into mice via the intramuscular route [56]. Recovery of adult worms from the mesenteries 4 weeks later was very low but was in agreement with other reports where newly transformed schistosomula were electroporated with dsRNAs prior to intramuscular injection into mice and subsequent recovery of adult worms from the mesenteries [41]. The natural route of *S. mansoni* infection is through percutaneous penetration of cercariae; exposure of laboratory mice to cercariae is generally performed via the abdomen or tail. Intramuscular injection of mice with schistosomula is not the natural infection route and consequently may have contributed to the low recovery rates. Despite the low recovery of adult parasites, we consistently over three experiments recovered significantly fewer worms from the mice injected with *tsp* dsRNA treated parasites. Moreover, *tsp* mRNA levels in those parasites that were recovered from mice were higher than levels in parasites cultured *in vitro* for the same time period after electroporation with dsRNAs, indicating that the parasites that survived *in vivo* had not succumbed to the effects of RNAi.

We envisage that interruption of *Sm*-TSP-1 and TSP-2 protein expression in the tegument of maturing schistosomula results in impaired turnover of the tegument apical membrane complex. Our observations from adults and schistosomula treated with *Sm-tsp-2* dsRNA would indicate that a likely role for *Sm-tsp-2* is in invagination and internalization of the surface membrane, and perhaps the closure and internalization of surface invaginations. This postulate is consistent with the suggestion that TSP-2 binds other parasite sub-surface and surface molecules in the tegument. The vaccine efficacy of TSP-2 may thus result from impairment of the surface recycling mechanisms in developing and adult schistosomes. While this impaired surface turnover was not deleterious to *in vitro* cultivated adult worms and schistosomula, the effect was particularly marked in treated schistosomula transferred into the host. In addition, schistosomes have the capacity to adsorb host blood molecules that mask antigenic epitopes from the host's immune system [7]. By affecting surface tegument development and turnover, suppression of *tsp* expression (and potential disruption of TEMs) may render the organism susceptible to immune recognition and clearance.

## Materials and Methods

### Ethics statement

All animals were maintained in accordance with the guidelines of the Animal Ethics Committee (AEC) of Queensland Institute of Medical Research and the Institutional Animal Care and Use Committee (IACUC) of The University of Pennsylvania. All studies and procedures were reviewed and approved by the AEC and IACUC of Queensland Institute of Medical Research and The University of Pennsylvania respectively.

### Parasites

The Puerto Rican strain of *S. mansoni* and *Biomphalaria glabrata* snails were provided by the National Institutes of Allergy and Infectious Diseases Schistosomiasis Resource Centre at the Biomedical Research Institute (Rockville, Maryland, USA). *B. glabrata* infected with miracidia were exposed to incandescent light for 1h to obtain cercariae which were used to percutaneously infect 6–8 week old C57BL/6 female mice (www.jax.org). After 8 weeks, adult parasites were recovered by hepatic-portal perfusion and then washed three times with wash medium containing RPMI 1640, 1% antibiotic/antimycotic and 10 mM Hepes (www.invitrogen.com) before experimentation.

To obtain schistosomula, cercariae were passed through a 22-gauge emulsifying needle 25 times to mechanically shear the cercarial tails from the bodies [57]. The resulting schistosomula were isolated from free tails by centrifugation through a 60% percoll gradient [58], washed three times with washing medium and incubated at 37°C under 5% CO_2_ atmosphere before experimentation.

### Immunofluorescent labelling of live schistosomula

Three hour schistosomula (n = 500) were blocked in blocking buffer containing 1% goat serum in Dulbecco's Phosphate Buffered Saline (DPBS) containing MgCl_2_ and CaCl_2_ (www.invitrogen.com). Schistosomula were labelled with sera against recombinant *Sm*-TSP-1, *Sm*-TSP-2 or control pre-vaccination sera [28] diluted to 1∶50 in blocking buffer for 1 h. Secondary goat anti-mouse Ig-FITC (www.chemicon.com) was then introduced at 1∶100 dilution in blocking buffer for 1 h followed by 4% paraformaldehyde to fix the parasites. Incubations were carried out at 4°C and parasites were washed in DPBS between incubations. Approximately 200 schistosomula were examined using a Leica MRIRB microscope and DC500 camera (www.leica.com).

### Synthesis of dsRNAs

dsRNAs were prepared from DNA templates that were amplified by PCR from *S. mansoni* paired adult worm cDNA using primers flanked with T7 RNA polymerase promoter sequence (underlined) at the 5′ ends. A 523 bp fragment of the *Sm*-*tsp-1* coding DNA was generated using primers (forward: 5′-TAATACGACTCACTATAGGGACTTGCTTCGGGACAACAAC-3′, reverse: 5′-TAATACGACTCACTATAGGGTTCGAAAGCTGCAATAGAAACA-3′) and a 565 bp fragment of the *Sm*-*tsp-2* coding DNA was produced using primers (forward: 5′-TAATACGACTCACTATAGGGTGATTGTGGTTGGTGCACTT-3′, reverse: 5′-TAATACGACTCACTATAGGGGACCAATGCGAACAGAAACA-3′). The GenBank accession numbers for *Sm*-*tsp-1* and *Sm*-*tsp-2* are AF521093 and AF521091, respectively. The PCR products were then utilized as templates for synthesis of dsRNAs using the T7 Megascript kit (www.ambion.com), following the manufacturer's instructions. An irrelevant negative control, firefly *luciferase* dsRNA derived from pGL3-basic (www.promega.com), was prepared as described previously [31].

### dsRNA delivery in schistosomes

Adult schistosomes were cultured *in vitro* in Medium 199 (www.invitrogen.com) supplemented with 10% fetal calf serum (www.gembio.com), 1% antibiotic/antimycotic and 10 mM Hepes at 37°C under 5% CO_2_ atmosphere. Five pairs of adult worms were soaked in the presence of *Sm*-*tsp-1*, *Sm*-*tsp-2* or *luciferase* dsRNAs at 1 µg/ml for 7 days with changes of media and dsRNAs every second day. Schistosomula were maintained at 37°C with 5% CO_2_ in Medium 169 [36] supplemented with 10% human AB serum (www.gembio.com) and mouse whole blood. Larval parasites (3 h old) were soaked in 1 µg/ml of *Sm*-*tsp-1*, *Sm*-*tsp-2* or *luciferase* dsRNAs and cultured *in vitro* at 37°C under 5% CO_2_ atmosphere for 7, 14 and 21 days, with fresh changes of media, blood and dsRNAs every second day. Adult and larval parasites were washed in wash medium prior to RNA or protein extraction.

### Infection of mice with dsRNA-treated schistosomula

Newly transformed schistosomula were incubated in wash medium at 37°C with 5% CO_2_ for 3 h. Parasites were then resuspended in 50 µl of wash medium with 100 µg/ml of *Sm*-*tsp-1*, *Sm*-*tsp-2* or *luciferase* dsRNAs and electroporated in a 4 mm cuvette at 125 V for 20 ms using a square-wave BTX ECM 830 electroporator (www.btxonline.com). After three washes in wash medium, schistosomula were counted and 2000 were injected intramuscularly into each C57BL/6 female mouse (3 mice per group) using a 23-gauge needle. Adult worms were perfused 28 days later to assess the number of worms that had matured and reached the mesenteries.

### Real-time quantitative RT-PCR

RNA was isolated from parasites using RNeasy Mini kit (www.qiagen.com) and then treated with Turbo DNA-free endonuclease (www.ambion.com) to remove contaminating genomic DNA. The quantity of RNA was measured on a Nanodrop Spectrophotometer (www.nanodrop.com) and 250 ng of total RNA, SuperScript II reverse transcriptase (www.invitrogen.com) and oligo dT_15_ primer (www.promega.com) were used to synthesize first strand cDNA.

The following primers were designed for real-time qRT-PCR; *Sm-TSP-1* (forward: 5′-TGGTTGTGCTTATTGGGTTG-3′ and reverse: 5′-TGATGTCTTGTGCCTCTGGT-3′); *Sm*-TSP-2 (forward: 5′-CGAAATTGAACCCCCACTAC-3′ and revere: 5′-CATGCTCCAAACATCCCTAAA-3′); *Sm*-Paramyosin (forward: 5′-CGTGAAGGTCGTCGTATGGT-3′ and reverse 5′-GACGTTCAAATTTACGTGCTTG-3′) and *Sm*-α-tubilin (forward: 5′-CCAGCAAAATCAGATGGTGAA-3′ and reverse: 5′-TTGACATCCTTGGGGACAAC-3′). qRT-PCR was conducted in triplicate and each reaction underwent 40 amplification cycles using an Applied Biosystems 7500 real-time PCR system (www.appliedbiosystems.com) with cDNA equivalent to 20 ng of total RNA, 50 nM of primers and SYBR green PCR Master Mix (www.appliedbiosystems.com). Dissociation curves were generated for each sample to verify the amplification of a single PCR product. *Sm-tsp* transcript levels were calculated relative to *Sm-paramyosin* in test and irrelevant dsRNA treated parasites using the 2^−ΔΔCt^ method [59], and data was expressed as percent differences. For relative endogenous expression of *tsp* mRNAs in schistosome life cycle stages, *Sm*-α-*tubulin* was used as the endogenous standard. *Sm*-*paramyosin* was used as the housekeeping gene for analyzing *Sm-tsp* expression in RNAi experiments.

### Evaluation of protein expression

RNAi-treated adult parasites and schistosomula were harvested after 7 days and then lysed with 1% Triton X-100 in Tris buffered saline supplemented with complete protease inhibitor cocktail EASYpacks (www.roche.com). Protein concentrations of lysates were determined using a BCA protein assay kit (www.pierce.com), and lysates were electrophoresed in 12% SDS-PAGE gels at concentrations of 2, 1, 0.5 and 0.25 µg total protein per well. Proteins were transferred to nitrocellulose membrane (Hybond-ECL, www.gehealthcare.com) and then probed with either anti-*Sm*-TSP-2 (3H5/2) monoclonal antibody supernatants (L. Cooper, M. Tran and A. Loukas, unpublished) diluted 1∶1,000 followed by anti-mouse Ig-HRP (www.chemicon.com) diluted 1∶5,000. Reactive proteins were detected by ECL (www.gehealthcare.com) as per the manufacturer's instructions. To assess equal protein loading, nitrocellulose membranes were stripped after reacting with anti-TSP-2 antibodies and then re-probed with anti-paramyosin (Sm4B1) monoclonal antibody supernatants [60] diluted at 1∶1,000 followed by anti-mouse Ig-HRP. Experiments were repeated three times and protein quantities in gel bands were determined using Syngene Tools and software (www.syngene.com).

### Electron microscopy

Adult parasites and schistosomula were soaked in 1 µg/ml of *Sm*-*tsp* or *luciferase* dsRNAs for 7 days at 37°C under 5% CO_2_ atmosphere, washed three times in wash medium and then fixed in 3% glutaradehyde in 0.1M phosphate buffer at pH 7.4, followed by fixation in potassium ferricyanide-reduced osmium tetroxide. After fixation, parasites were dehydrated in acetone and embedded in Epon Resin (ProSciTech). Ultrathin sections were mounted onto copper grids, contrasted in uranyl acetate and lead citrate and examined and photographed using a JEM 1011 transmission electron microscope operated at 80 kV and equipped with a digital camera.

A morphometric approach was employed to quantify possible changes to tegument structure in schistosomula treated with *Sm-tsp-2* relative to those treated with *luciferase* dsRNA. Point counting stereology [61], [62] was used to measure the volume of tegument occupied by vacuolar compartments or tegument invaginations in the tegument. Such regions were evident as clear spaces in TEM sections. Twenty individual schistosomula were selected at low magnification in the TEM. For each parasite, the first region of tegument observed that fulfilled the two criteria below was photographed at ×10,000 magnification. Criteria for selection were, firstly, that the region photographed was from the lateral aspect of a parasite that was clearly longer than wide and in which internal organs were present, and secondly, that the region was not excessively spinous. Volume density of vacuolar compartments of tegument were estimated using grids generated by Image J analysis software (NIH Besthesda), and were calculated as the number of points on the grid intersecting a vacuolar space divided by the number of points intersecting the tegument. This was measured across the entire profile of the tegument in each electron micrograph, so that only one measure was obtained for each schistosomulum. In addition to the volume density measure, the thickness of the tegument was measured at 10 different points using the line tool in Image J. For each measure, a line was drawn digitally on each micrograph from the basal membrane of the tegument to the apical membrane. Regions where the tegument was excessively invaginated, and those containing isolated spines and sensory receptors were not measured. The 10 thickness measurements were averaged for each schistosomulum.

### Statistical analyses

All data are presented as the mean±standard error. Differences between groups were assessed for statistical significance using Student t-test (GraphPad Prism Software, www.graphpad.com). A statistically significant difference for a particular comparison was defined as p<0.050.
